# 3D Tracking of Human Motion Using Visual Skeletonization and Stereoscopic Vision

**DOI:** 10.3389/fbioe.2020.00181

**Published:** 2020-03-05

**Authors:** Matteo Zago, Matteo Luzzago, Tommaso Marangoni, Mariolino De Cecco, Marco Tarabini, Manuela Galli

**Affiliations:** ^1^Department of Electronics, Information and Bioengineering, Polytechnic of Milan, Milan, Italy; ^2^Department of Mechanical Engineering, Polytechnic of Milan, Milan, Italy; ^3^Department of Industrial Engineering, University of Trento, Trento, Italy

**Keywords:** movement measurement, gait analysis, computer vision, artificial intelligence, markerless motion capture

## Abstract

The design of markerless systems to reconstruct human motion in a timely, unobtrusive and externally valid manner is still an open challenge. Artificial intelligence algorithms based on automatic landmarks identification on video images opened to a new approach, potentially e-viable with low-cost hardware. OpenPose is a library that t using a two-branch convolutional neural network allows for the recognition of skeletons in the scene. Although OpenPose-based solutions are spreading, their metrological performances relative to video setup are still largely unexplored. This paper aimed at validating a two-cameras OpenPose-based markerless system for gait analysis, considering its accuracy relative to three factors: cameras' relative distance, gait direction and video resolution. Two volunteers performed a walking test within a gait analysis laboratory. A marker-based optical motion capture system was taken as a reference. Procedures involved: calibration of the stereoscopic system; acquisition of video recordings, simultaneously with the reference marker-based system; video processing within OpenPose to extract the subject's skeleton; videos synchronization; triangulation of the skeletons in the two videos to obtain the 3D coordinates of the joints. Two set of parameters were considered for the accuracy assessment: errors in trajectory reconstruction and error in selected gait space-temporal parameters (step length, swing and stance time). The lowest error in trajectories (~20 mm) was obtained with cameras 1.8 m apart, highest resolution and straight gait, and the highest (~60 mm) with the 1.0 m, low resolution and diagonal gait configuration. The OpenPose-based system tended to underestimate step length of about 1.5 cm, while no systematic biases were found for swing/stance time. Step length significantly changed according to gait direction (*p* = 0.008), camera distance (*p* = 0.020), and resolution (*p* < 0.001). Among stance and swing times, the lowest errors (0.02 and 0.05 s for stance and swing, respectively) were obtained with the 1 m, highest resolution and straight gait configuration. These findings confirm the feasibility of tracking kinematics and gait parameters of a single subject in a 3D space using two low-cost webcams and the OpenPose engine. In particular, the maximization of cameras distance and video resolution enabled to achieve the highest metrological performances.

## Introduction

The measurement of human motion represents one of the most interesting and challenging topics of metrology. Optical motion tracking solutions can be broadly categorized into marker-based and markerless systems (Winter, 1990; Zhou and Hu, 2008). Mostly represented by the first group, the modern technological standards ground on established measurement principles and techniques: the position of joints and the orientation body segments are obtained through the three-dimensional localization of passive (or less often, active) markers, fixed on subjects' body and captured by a calibrated multi-camera stereophotogrammetric video system (Cappozzo et al., 2005). The human body is a complex, self-occluding and only partially rigid entity (Mündermann et al., 2006). Thus, instead of directly tracking human body pose, these systems work by identifying common object features in consecutive images (fiducial points or landmarks), which are used to track the motion of a series of rigid bodes connected by rotational joints (Winter, 1990). This solution provides the best metrological performances, in terms of accuracy in the markers' localization (usually in the order of 10ths of millimeters), repeatability and frequency of measurements (Ma'touq et al., 2018). Owing to their cost, complexity and required personnel to run the recording and place the markers on specific anatomical landmarks, marker-based systems are mainly used in specialized laboratories for clinical/rehabilitation applications or entertainment and digital animation (Winter, 1990; Cappozzo et al., 2005).

With the aim of limiting these drawbacks, in the last decades the interest toward markerless solution has grown rapidly, trying either to reduce the cost of technology or to simplify the process (Abbondanza et al., 2016; Ronchi and Perona, 2017; Colyer et al., 2018; Mizumoto et al., 2018; Tanaka et al., 2018; Tarabini et al., 2018a; Clark et al., 2019). Markerless systems are based on four main components, namely a camera system, a body model, the image features used and the algorithms that determine shape, pose and location of the model itself (Colyer et al., 2018). Two families of camera systems can be used, differing by whether or not they produce a so-called “depth map,” i.e., an image where each pixel describes the distance of a point in the scene from the camera. Probably the best-known depth-sensing camera systems (often referred to as RGB-D cameras as they capture both color and depth) are Microsoft Kinect, Intel Realsense, and StereoLabs Zed. These solutions are particularly effective for real-time full body pose estimation in interactive systems and videogames (Shotton et al., 2011; Ye et al., 2013), but they also have limitations that hinder their wide application in clinical or biomechanical setting: short-range, inoperability in bright sun light, and potential interference between multiple sensors (Colyer et al., 2018). In addition, the accuracy in motion tracking is still lower than marker-based systems, which actually remain the gold standard.

Recently, novel artificial intelligence algorithms based on automatic landmarks identification on video images (computer vision) opened to a new approach for markerless motion capture, which became potentially feasible with low-cost hardware (Cao et al., 2016; Colyer et al., 2018; Clark et al., 2019). In that, machine learning techniques were exploited to identify the nodes of a skeletal structure describing the posture of a human subject within a given image frame. As the associated computational burden made this method practicably unviable, the process was optimized by a research group from the Carnegie Mellon University, who released a processing framework called *OpenPose* (Cao et al., 2016). OpenPose takes as input color images from simple web-cameras and using a two-branch convolutional neural network (CNN) produces as output confidence maps of keypoints, and affinity for each keypoint pair (that is, belonging to the same skeleton). This way, OpenPose allows for the recognition of skeletons of multiple persons in the same scene. Some Authors adopted these OpenPose-based solutions as a functional block of their research: an example is Huang et al. (2017), in which OpenPose was used as an initialization step for the reconstruction of 3D human shape; a different approach is presented in Mehta et al. (2017), in which a 3D skeletal model was obtained starting from a single planar image.

Although promising results were obtained, the design of markerless systems able to reliably reconstruct human motion in a timely, unobtrusive and externally valid manner is still an open challenge (Colyer et al., 2018). Among the fast-growing studies on the application to various case studies, only a few focused on the accuracy of subjects' three-dimensional reconstruction: the performance of OpenPose in the computation of the lower limbs angles were analyzed with a single camera (Gu et al., 2018), and compared to a multi-camera marker based system. However, to the best of our knowledge, a targeted metrological characterization of data processing with multiple viewpoints is still missing in the case of automated walking analysis. At present, example of OpenPose applications for the extraction of gait parameters are scant. We hypothesize that the cameras resolution and positioning, as well as the walking direction (i.e., angle with respect to cameras) could affect the accuracy and thus feasibility of such systems in the clinical setting. Thus, this paper aims at describing and validating an OpenPose-based markerless motion tracking system for gait analysis against a gold-standard commercial marker-based motion capture system, discussing the extent to which the aforementioned factors affect the tracking quality.

## Methods

### Experimental Design and Participants

This observational case-series study was designed to determine the metrological performance of the stereoscopic system featured by OpenPose. The study involved two healthy volunteers who performed a walking test at comfortable walking speed within an instrumented gait analysis laboratory. The two participants were both 24-years-old male adults, with the following heights and body masses: 1.73 m and 61 kg, 1.82 m and 75 kg. They wore minimal, close-fitting clothes. Participants were instructed about the aims and benefits of the study, and they both signed a written informed consent prior to laboratory sessions. As this study did not involve any clinical intervention or functional/physical evaluation, the approval from the Ethics Committee was not required. The study was carried out in accordance with the 1964 Helsinki declaration and its later amendments.

The effect of three factors potentially influencing the accuracy of the proposed system were considered:

1. Cameras' relative distance: cameras were positioned 1 m and then 1.8 m apart;
2. Gait direction, straight or diagonal, defined by means of visual references positioned along the path (the same for all the tests repetitions). In the second, additional sources of error may arise from the occlusions between body parts; subjects walked on a footboard and the walking direction was perpendicular to the cameras' connecting axis.
3. Video resolution: high (1,312 × 736 pixel), and standard (640 × 480 pixel). Both resolutions were obtained by scaling the camera native resolution with a cubic interpolation, this way we avoided the repetition of recording sessions.

Given that each factor assumed two levels, 2^3^ (8) configurations were possible. Each test configuration was replicated 3 times per each volunteer; 48 tests were therefore performed.

### Measurement System and Equipment

Two full-HD webcams (PC-W1, Aukey, Shenzhen, China) with a native image resolution of 1920 × 1080 pixels and a 1/2.7” CMOS sensor were used. Cameras acquired images at 30 Hz, with contrast and brightness automatically selected by the software provided by the manufacturer. Cameras were fastened on an aluminum bar perpendicular to the strait gait direction at a height of 2.3 m, framing the subject frontally.

**Figure 1:**
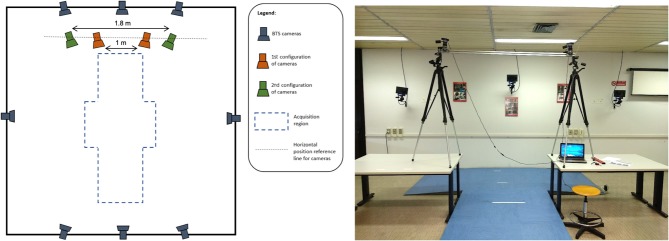
Laboratory setup, schematic **(left)** and pictorial **(right)** view.

A stereophotogrammetric motion analyser (Smart-D, BTS Bioengineering, Milano, Italy) equipped with eight infrared cameras sampling at 100 Hz was used as reference measurement system. The system was calibrated according to the manufacturer's specification, and the error in markers' location reconstruction was 0.2 mm on a working volume of 3 × 2 × 2 m. Figure 1 shows the implemented measurement infrastructure.

### Procedures

**Figure 2:**
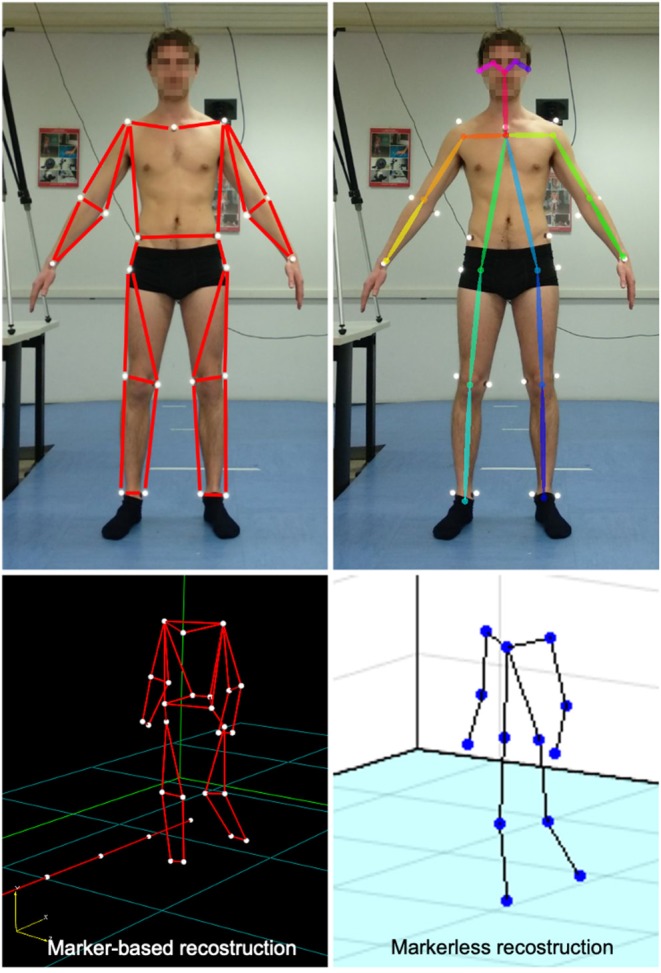
Stick diagrams as returned by the marker-based optical system (top, left) and OpenPose model (top, right); corresponding 3D reconstruction of the skeletal structures during walking (bottom).

The measurement process can be summarized as follows:

1. Calibration of the stereoscopic system using planar patterns (Zhang, 2000; Hartley and Zisserman, 2003). Cameras calibration was performed within Matlab (v2018b, The Mathworks Inc., Natwick, USA) by means of the Camera Calibration Toolbox. A black and white checkerboard whose geometry is known (70 × 50 cm) is framed by the two cameras while spanning the checkerboard into the working volume. The Toolbox returns an estimate of the cameras internal and external parameters (i.e., lens distortion, camera relative orientation and position). To get a calibration metric, the reprojection error is computed by projecting the checkerboard points from world coordinates into image coordinates. Mean reprojection error was 0.18 pixels in the 1-m distance configuration, and 0.12 pixels in the 1.8 m configuration.
2. Acquisition of two video recordings, *a* and *b* (one per each webcam), using the cameras of the stereoscopic system. Each recording allowed to collect between four and five steps, according to the laboratory dimension, and lasted 4.5–6.5 s.
3. Simultaneous recording using the reference, marker-based optical system. Twenty-four reflective markers were placed on the subject in the following anatomical landmarks (see Figure 2): sternum and sacrum; right and left acromion, medial and lateral humeral epicondyles, radius and ulnar styloid process, antero-superior iliac spines, greater trochanter, medial and lateral femoral epicondyles, medial and lateral malleoli. This marker set was adapted from standard protocols used in clinical gait analysis (Davis et al., 1991; Zago et al., 2017), and was designed to match the skeletal configuration of OpenPose (Figure 2). To do so, wrists, elbows, knees and ankles joint centers were located at the midpoint (average) of medial and lateral markers. Hip joint centers were computed using regression equations as prompted by the International Society of Biomechanics standards (Wu and Cavanagh, 1995).
4. Video processing within OpenPose to extract the skeleton *S* of a (single) subject in each video recording (*S*_*a*_ and *S*_*b*_).
5. Synchronization of the two videos (see paragraph Data Synchronization and Spatial Alignment).
6. Triangulation of the skeletons *S*_*a*_ and *S*_*b*_ using the calibration outcome (step 1) to obtain the three-dimensional coordinates of the joints and alignment between coordinate system of step 4.
7. Computation of gait parameters (see paragraph Target Parameters Computation) based on the three-dimensional coordinates obtained.
8. Evaluation of the OpenPose accuracy for each single test according to the metrics defined in the following paragraph.
9. Evaluation of the dependence of accuracy from the factors' levels using a 3 × 2 Analysis of Variance.

### Data Processing

A set of 18 2D keypoints coordinates for body pose estimation (in pixels) are returned by OpenPose from video images; 2D keypoints are located in relevant body landmarks (such as left hand, right hand, face, etc.) and were used to determine the 3D Cartesian coordinates, positioning the skeletal model of the subject in the space with respect to reference system of one camera. This operation was performed using the Matlab Computer Vision System Toolbox (v2018b, The Mathworks Inc., Natwick, USA), obtaining the 3D stereoscopic triangulation of the camera pixel coordinates, which included:

- the intrinsic calibration parameters of each camera, for the assessment of focal lengths, camera centers and distortion parameters;
- the extrinsic calibration parameters, accounting for the relative position of cameras;
- the undistortion of pixel coordinates;
- the application of a functional triangulation for each of the 2D keypoints for the identification of the corresponding 3D coordinates in the epipolar plane.

The resulting output was the 3D skeletal model of the subject, as shown in the bottom-right panel of Figure 2.

Prior to further processing, coordinates returned by both the OpenPose and the marker-based reference system were filtered using a zero-lag, 2nd order Butterworth filter with a cut-off frequency of 10 Hz.

#### Data Synchronization and Spatial Alignment

Since a physical trigger for the synchronization of the cameras with the motion capture system was not available, we asked the subjects to perform a sequence of repeated actions (to beat the right hand on the right hip). The synchronization procedure was repeated before each single test and it was achieved by overlapping the time series of the distance between the right wrist and right hip markers returned by the two systems. Prior to do so, the signals were both downsampled (cubic splines interpolation) to 30 Hz. Drift errors due to different sampling rates (100 Hz for the marker-based system, 30 Hz for the webcams) were negligible given the test duration of a few seconds.

The spatial alignment of the reference systems completed the measurement systems calibration: the 3D coordinates provided by the triangulation of OpenPose data were originally expressed in a reference system located in the optical center of one of the cameras, oriented as the camera itself. The marker-based system returns 3D coordinates resolved in global (laboratory) reference system fixed on the ground at the center of the working volume. These two coordinate systems were moved to a new, coincident, reference frame, positioned midway between the two cameras, with the origin at the ground level and with the axes directed as those of the original marker-based system. The alignment procedure was taken from Kabsch (1976) and involved the initial rotation of the OpenPose reference system, followed by the translation toward the desired origin.

#### Target Parameters Computation

**Figure 3:**
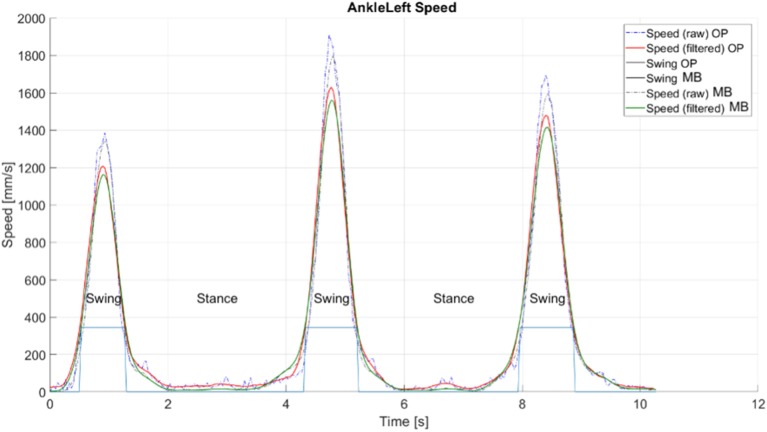
Extraction of gait phases from the trajectories of ankle nodes' velocity, explanatory example taken from a straight gait test. OP, OpenPose-based system; MB, marker-based optical system.

Within the OpenPose-based system, the definition of the gait phases relies on the recognition of the foot condition—stance or swing (Saggin et al., 2013). The distance between two successive stance statuses represents the target measurement. In our case, the processing structure was taken from Tarabini et al. (2018b) and involved the analysis on the velocities of the nodes located at the ankle level (Figure 3). Given a window of *n* elements, the magnitude of the velocity (*v*) of the two ankle nodes was computed as:

$$\begin{array}{l} v=f\cdot \sqrt{\overset{n}{\underset{i=1}{\prod }}{\left(x_{i}-x_{i-1}\right)}^{2}+\overset{n}{\underset{i=1}{\prod }}{\left(y_{i}-y\right)}^{2}+\overset{n}{\underset{i=1}{\prod }}{\left(z_{i}-z_{i-1}\right)}^{2}} \end{array}$$

where *f* is the sample frequency (30 Hz). To minimize the influence of noise and ease foot status detection, a moving average lowpass filter was then applied on *v*, with a period of 12 samples (with a 30 Hz sampling frequency, the first zero of the filter transfer function is at 1.25 Hz). Two thresholds on the filtered velocity signal of the ankle node were set for the identification of the foot status: *HystLowSpeed* and *HystHighSpeed*. These were automatically obtained for each subject from a complete gait test. After an initial sorting of all velocities assessed from the test and reorganized in the form of a histogram, the values were computed as:

- *HystHighSpeed*: upper threshold limit, as the value corresponding to 65% of the sorted velocities: when the joint's filtered speed was higher than this value, then the foot was considered in the swing state (1).
- *HystLowSpeed*: lower threshold limit, equal to 80% of *HystLowSpeed*, to avoid erroneous swing's end caused by small variations induced by residual noise components. In this case, the foot was considered in the stance state (0).

To get correct gait parameters' values, it is essential to consider complete steps only. For such a reason, four cases were analyzed:

1. Foot enters the considered acquisition window in swing state (1) and exits still in swing state (1) (if the acquisition contained at least a complete step, first and last step were not considered).
2. Foot enters the considered acquisition window in stance state (0) and exits in swing (1) state (the last step was not considered).
3. Foot enters the considered acquisition window in swing state (1) and exits in stance state (0) (the first step was not considered).
4. Foot enters in the considered acquisition window in stance state (0) and exits still in stance state (0) (if the acquisition contained any number of steps, they were all considered).

### Evaluation of Accuracy

In each test, the accuracy of the proposed system was evaluated in terms of two sets of parameters, retrieved from the same recorded dataset:

- **Error in the reconstruction of the trajectories**, computed as the Root Mean Square (RMS) distance between the trajectories of selected, corresponding skeletal nodes. In doing so, the most similar physical fiducial points were considered: wrists, elbows, knees and ankles. Indeed, the reference and the proposed skeletal structures do not correspond perfectly. Thus, a minimization procedure was used to align the thirteen landmarks of the skeleton, and a roto-translation of the trajectories obtained with the OpenPose-based system was performed to align them to the correspondent reference (marker-based coordinates). The complete procedure is described in Abbondanza et al. (2016) and Tarabini et al. (2018b) and it is based on the calculation of the Euclidean distance in each frame between correspondent keypoints of the two systems. This method was already used to synchronize trajectories acquired with different measurement systems, and it proved to be unbiased also in presence of offset between the skeletal markers (Abbondanza et al., 2016; Tarabini et al., 2018b).
- **Error in gait space-temporal parameters**: step length (distance between consecutive heel-strikes position), stance and swing time were extracted (Perry and Burnfield, 2010). The RMS error with the correspondent parameters computed with the reference marker-based system was computed.

### Statistical Analysis

The effect of the three factors (cameras' distance, gait direction, and resolution) on the measurement error was assessed using the following analysis of variance (ANOVA) model design (Moschioni et al., 2013):

$$\begin{array}{l} \xi =\beta_{0}+\beta_{1}x_{1}+\beta_{2}x_{2}+\beta_{3}x_{3}+\beta_{\left(1,2\right)}x_{1}x_{2}+\cdots +\epsilon \end{array}$$

where ξ is the dependent variable, namely the skeletal node position error (RMS) or the error of one of the gait analysis parameters (step length, stance and swing time), and *x*_*i*_ are the independent variables (previously referred to as factors). β_0_ is the global tests average, β_*i*_ and β_(1,2)_ are used to describe the effect of the independent variables and their interactions (in particular, gait direction × camera distance interaction was assessed); ϵ is the residual, namely the difference between the actual data behavior and the model prediction. A significance alpha level of 5% was implemented throughout.

In addition, Bland-Altman plots were used to graphically compare gait analysis parameters obtained with the reference and OpenPose-based systems.

## Results

**Figure 4:**
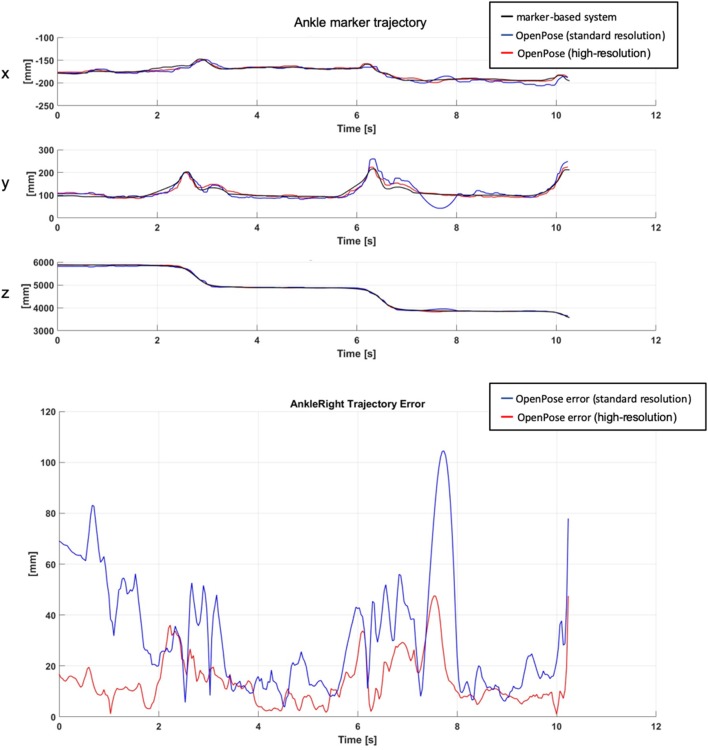
Sample trajectories of a landmark (position of the right ankle) obtained from the reference marker-based (black) and markerless, OpenPose-based (blue and red) systems **(top)**; corresponding RMS distance **(bottom)**.

**Figure 5:**
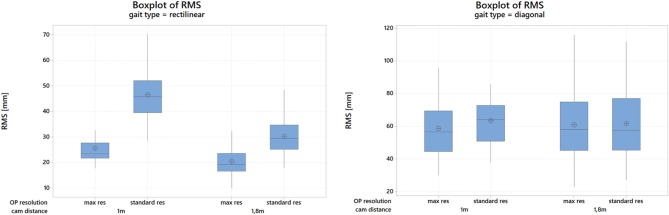
Boxplots of the RMS distance (measurement error) for the straight **(left)** and diagonal **(right)** walking tests; cam: cameras, res: resolution; OP, OpenPose.

**Figure 6:**
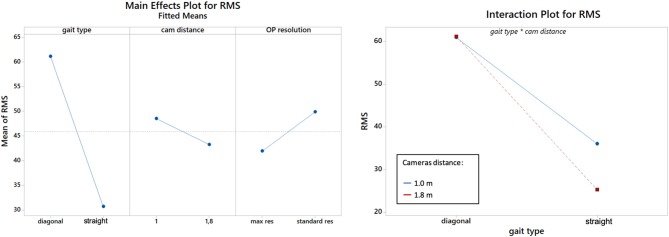
Effects plot for the RMS distance, according to the gait type (walking direction), cameras distance and resolution **(left)**. Gait type×distance interaction plot **(right)**. Cam: cameras, res: resolution; OP, OpenPose.

Figure 4 shows an explanatory plot of the original joints coordinates and of the corresponding measurement error over time. Overall measurement errors (RMS) are reported in Tables 1, 2, and graphically summarized in the boxplots of Figure 5; Table 3 displays the relevant statistic: all factors (*p* < 0.01) and interaction (*p* < 0.001, see Figure 6) resulted to be statistically significant relative to trajectories reconstruction error. The lowest error (about 20 mm) was obtained with the 1.8 m, highest resolution and straight gait configuration, and the highest (>60 mm) with the 1.0 m, low resolution and diagonal gait configuration.

**Table 1:** Root Mean Square errors measured at different skeleton nodes as a function of gait direction, camera distance and video resolution.

**#**	**Gait type**	**Straight gait**				**Diagonal gait**			
	**Distance (m)**	**1.8**	**1.8**	**1.0**	**1.0**	**1.8**	**1.8**	**1.0**	**1.0**
	**Resolution**	**LR**	**HR**	**LR**	**HR**	**LR**	**HR**	**LR**	**HR**
1	Sternum	25.2	16.8	41.1	22.1	65.8	54.0	69.5	60.1
2	Shoulder, left	32.3	20.0	42.2	26.5	46.2	45.5	52.8	51.2
3	Shoulder, right	27.5	17.7	37.2	22.1	42.1	39.2	48.3	41.2
4	Elbow, left	28.2	18.3	49.8	24.6	69.9	61.6	69.1	58.3
5	Elbow, right	27.2	18.8	50.3	27.9	62.7	74.4	48.5	44.6
6	Wrist, left	23.3	17.1	45.1	22.9	51.6	51.8	79.1	54.0
7	Wrist, right	25.4	16.1	48.6	22.3	65.1	66.9	56.1	48.2
8	Hip, left	33.0	21.9	48.5	29.0	79.9	81.1	67.5	75.7
9	Hip, right	34.6	23.4	50.1	41.4	79.5	82.5	71.6	73.5
10	Knee, left	30.9	23.4	60.0	29.2	53.7	53.0	58.7	55.4
11	Knee, right	33.9	24.8	39.6	19.9	58.4	57.6	63.7	48.3
12	Ankle, left	39.5	26.1	62.2	24.0	69.1	68.7	87.4	103.3
13	Ankle, right	35.8	26.2	34.5	21.4	63.5	62.7	64.9	61.3
-	Mean	30.5	20.8	46.9	25.6	62.1	61.5	64.4	59.6
-	SD	4.6	3.5	7.9	5.4	11.1	12.6	11.1	16.0

*Values in mm*.

**Table 2:** Root Mean Square errors measured between the reference (marker-based) and OpenPose-based systems on selected spatio-temporal gait parameters.

**Gait type**	**Straight gait**				**Diagonal gait**			
**Distance (m)**	**1.8**	**1.8**	**1.0**	**1.0**	**1.8**	**1.8**	**1.0**	**1.0**
**Resolution**	**LR**	**HR**	**LR**	**HR**	**LR**	**HR**	**LR**	**HR**
Step length (cm)	3.26	1.53	7.42	1.93	2.45	1.66	3.25	1.23
Swing time (s)	0.04	0.05	0.05	0.02	0.03	0.03	0.03	0.06
Stance time (s)	0.05	0.05	0.05	0.05	0.07	0.07	0.05	0.08

**Table 3:** Statistical outcomes from the ANOVA computed on trajectories' RMS and on gait spatio-temporal parameters Root Mean Square errors.

**Variable**	**Gait direction**		**Camera distance**		**Resolution**		Direction * distance	
	**F**	**p**	**F**	**p**	**F**	**p**	**F**	**p**
RMS	392.39	**<0.001**	8.11	**0.005**	44.5	**<0.001**	19.6	**<0.001**
Step length	7.84	**0.008**	5.84	**0.020**	28.46	**<0.001**	6.09	**0.018**
Stance time	1.45	0.235	3.24	0.079	4.57	**0.038**	0.29	0.591
Swing time	10.55	0.002	0.06	0.811	1.22	0.275	0.05	0.817

*Statistically significant values are in bold*.

**Figure 7:**
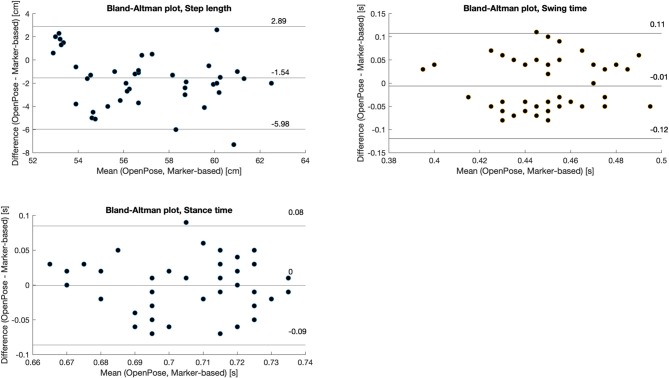
Bland-Altman plot of the pooled (selected) gait parameters, comparison between the OpenPose- and the marker-based systems.

Bland-Altman plots displaying gait parameters comparison are shown in Figure 7: the proposed system tended to underestimate step length of about 1.5 cm, while no systematic biases were found for swing/stance time. Step length significantly changed according to gait direction (*p* = 0.008), camera distance (*p* = 0.020) and resolution (*p* < 0.001, see Table 3). Consistently with trajectories' RMS, the lowest error in step length (1.53 cm) was obtained with the 1.8 m, high resolution and straight gait configuration. Among stance and swing times, only for the first emerged a significant factor, i.e., camera distance (*p* = 0.038), and the lowest errors (0.02 s and 0.05 s for stance and swing, respectively) were obtained with the 1 m, high resolution and straight gait configuration.

## Discussion

The findings of this work confirm to the feasibility of tracking kinematics and gait parameters of a single subject in a 3D space using two low-cost cameras and the OpenPose engine. The accuracy of markerless motion tracking depends on three factors: the occlusions between body parts, cameras position/orientation and video resolution; considering the best combination of the considered factors (cameras distance 1.8 m, maximum resolution, and no occlusions due to straight walking) the lowest error in 3D trajectories reconstruction was about 20 mm, the lowest error in swing/stance time was 0.03 s and 1.23 cm in step length. Values are comparable with intra-subject variability in clinical gait analysis investigations (Ciprandi et al., 2017; Temporiti et al., 2019), thus encourage a preliminary adoption of OpenPose-based markerless solutions in this setting. However, it should be noticed that a different configuration (smaller camera distance, lower resolution or diagonal gait direction) can negatively affect the results.

### Accuracy

In our optimal configuration, average markers RMS was about 20 mm. This can be considered a notable result, as it is only slightly higher than the error reported in a previous study (about 15 mm), where however eight cameras (fs = 120 Hz) and a subject-specific, way more complex anatomical model were used (Corazza et al., 2010). Dunne et al. reported an error of ~50 mm in reconstructing foot contact position with a single camera system (Dunn et al., 2014).

While several studies compared the outcome of an OpenPose markerless system to a traditional marker-based one (Clark et al., 2019), the majority focused on joint angles (Colyer et al., 2018) and to the best of our knowledge, none of them provided gait analysis parameters. Thus, a direct evaluation of the performances of our system is not straightforward. As a reference, Kinect-based markerless systems returned a lower accuracy of 2.5–5.5 cm in step length and a slightly lower accuracy of 60–90 ms in stance/swing time (Latorre et al., 2018). Previously, Barone et al. obtained comparable or slightly better results (accuracy of 3.7 cm for step length and 0.02 s for step duration) but they combined a markerless system with the signal coming from the accelerometer embedded in a smartphone (Barone et al., 2016).

The resolution of the stereoscopic system is not constant, being dependent on the physical distance between the subject and cameras. The method performances worsen as the subject distance from the sensor increases: errors presented in these work are average values, both summarizing the ideal situation in which the subject is filling the two image planes and the situation in which the subject is far from the camera with a less favorable optical sensor resolution.

### Effect of Camera Setup

Occlusions represented the most detrimental factor emerging from the comparison between gait types. The accuracy of results obtained in the diagonal gait tests was always lower than those obtained with a straight gait. In the 3D reconstruction all the other factors are almost negligible when occlusions are present. When body parts are occluded, OpenPose provides an estimation of the hidden landmarks, introducing an error that propagates in the 3D reconstruction. The problem is common with all the vision-based measurement systems and can be solved using more than 2 cameras simultaneously (most optoelectronic systems use from 6 to 12 cameras) so that each marker or joint is seen from more than 2 sensors.

Increasing the camera distance (from 1.0 to 1.8 m) in straight gait tests improved the accuracy of the reconstruction by 22.5%. Cameras relative distance and orientation influences the uncertainty of the triangulation, affecting the dimensions of the volume where the triangulated point can be placed. By positioning the two cameras further apart, the framed person is seen from a different perspective and the cameras are more convergent. This leads to a decrease of the capture volume where the triangulated point can be placed, but also a lower uncertainty in the triangulation process. In short, the higher the cameras distance, the narrower the working volume—but characterized by a higher accuracy.

When increasing video resolution, the error decreased of about 46% (1.0 m camera distance) and 32% (1.8 m camera distance). By increasing the video resolution, the uncertainty in the identification of the landmarks coordinates on the 2D images decreases, and the 3D reconstruction results more accurate. This comes at a cost: the main drawbacks are either higher processing time, to a first approximation linearly dependent to the number of pixels in the image, and more expensive hardware required to data processing. The spatial resolution of the system is not constant in the observed volume: the pixel to distance conversion factor depends on the position of the subject with respect to the cameras; consequently, the optical resolution worsen when the subject is far and occupies a small portion of the image. The problem can be solved by putting more cameras surrounding the subject and observing the motion from different directions, as in common optoelectronic systems. Since in our test the subject distance from the cameras varied approximately between 2 and 6 m, errors' numerical values are the average between optimal conditions (in which the subject fills the image) and worst ones. Consequently, in static applications when the subject is not moving, we can expect better performances with respect to values reported here.

### Limitations and Perspectives

This pilot study was limited to two healthy subjects; a larger population could be considered in further research to address, for instance, the effect of body size on the tracking accuracy, as well as potential effects of clothes. It is advisable that future research lines address the metrological characterization of multi-camera systems, which will enable a complete 360 degrees view of the subject. In this, the occurrence of occlusions will be minimized, and a more accurate reconstruction is expected, at the expenses of a more complex hardware infrastructure.

## Conclusion

In this work, a metrological characterization of OpenPose processing in the context of gait analysis by mean of low-cost stereoscopy was presented. Intentionally, no changes were made to the original software interface, working instead on the test configuration and on the influence factors in the metrological setup. Thus, all the insights concern the actual processing algorithms, not considering improvements deriving from the optimization or tuning of the code for a specific task.

Although future improvements in OpenPose performance are expected, both in terms of accuracy in landmarks identifications and processing speed, the proposed analysis considered general, “external” factors that will remain practically valid. In particular, we showed that the maximization of cameras distance and video resolution enabled to achieve the highest metrological performances. Therefore, system accuracy could further be improved by reducing the presence of occlusions not only through a better joint location prediction in the source images, but also multiplying the number of cameras, thus obtaining a perspective closer to the straight walking condition.

This work points the way to further applications in environments where a video-based acquisition would be particularly useful, i.e., those where a quick and economical evaluation by non-expert operators is required.

## Data Availability Statement

The datasets generated for this study are available on request to the corresponding author.

## Author Contributions

ML, TM, MZ, and MD carried out the experiment and undertook the data analysis. MZ and MT wrote the manuscript. MG and MT supervised the multidisciplinary project and revised the manuscript.

### Conflict of Interest

The authors declare that the research was conducted in the absence of any commercial or financial relationships that could be construed as a potential conflict of interest.
